# Treatment effects of fingolimod in multiple sclerosis: Selective changes in peripheral blood lymphocyte subsets

**DOI:** 10.1371/journal.pone.0228380

**Published:** 2020-02-03

**Authors:** Maria Hjorth, Nicolae Dandu, Johan Mellergård

**Affiliations:** 1 Department of Clinical Immunology and Transfusion Medicine, and Department of Biomedical and Clinical Sciences, Linköping University, Linköping, Sweden; 2 Department of Neurology in Linköping, and Department of Biomedical and Clinical Sciences, Linköping University, Linköping, Sweden; Purdue University, UNITED STATES

## Abstract

**Background:**

Treatment with fingolimod reduces inflammation in multiple sclerosis (MS) by inhibiting lymphocyte egress from lymph nodes. We aimed to map, in detail, the alterations in peripheral blood lymphocyte subpopulations in relation to clinical outcome in MS patients treated with fingolimod.

**Methods:**

Paired blood samples from relapsing-remitting MS patients (n = 19) were collected before and after one year of treatment with fingolimod (0.5 mg/day). Absolute counts and relative proportions of a broad set of T- B- and NK-cell subsets were analyzed by flow cytometry. Blood samples from 18 healthy controls were used for baseline comparisons.

**Results:**

Treatment with fingolimod markedly decreased the absolute numbers of all major lymphocyte subsets, except for NK cells. The reduction was most pronounced within the T helper (Th) and B cell populations (p<0.001). By phenotyping differentiation status of T cells, dramatic reductions within the naïve and central memory (CM) cell populations were found (p<0.001), while a less pronounced reduction was observed among effector memory (EM) cells (p<0.001). The numbers of regulatory T cells (Tregs) were also decreased (p<0.001), but to a lesser extent than other T cell populations, resulting in a relative preservation of Tregs with a memory phenotype (p = 0.002).

**Conclusions:**

Our results confirm that fingolimod therapy markedly reduces lymphocyte counts in peripheral blood of MS patients. Subgroup analysis of T cells showed that naïve and CM Th cells were the most profoundly affected and that memory Tregs were relatively preserved.

## Introduction

Multiple sclerosis (MS) is an inflammatory demyelinating disease of the central nervous system (CNS). T cells, B cells, and probably also autoantibodies are important factors contributing to the immunopathogenesis in MS patients [1–3]. Several disease-modifying treatments (DMTs) are available with the potential to reduce disease activity and improve the clinical course by modulating or suppressing the immune system in different ways. Natalizumab, for example, decreases the number of immune cells present in the CNS by blocking their migration from the peripheral circulation to the CNS, thereby increasing lymphocyte numbers in the circulation [4, 5]. Fingolimod represents another immunomodulatory principle that reduces the number of lymphocytes in the peripheral circulation. Fingolimod (Gilenya®) was the first oral DMT approved in MS [6–8]. The active form, fingolimod-phosphate, is a sphingosine 1-phosphate receptor (S1PR) modulator, which internalizes and degrades S1PR receptors [9, 10]. This leads to a significant impairment on lymphocyte recirculation since the S1PR is needed for egress of lymphocytes from the lymph nodes. Treatment with fingolimod confers profound alterations of the lymphocyte populations in peripheral blood and lymphoid compartments. A 70% reduction of the number of circulating lymphocytes has been reported in patients treated with fingolimod [8].

The massive impact of immunomodulation on the numbers and distribution of immune cells constitute a potential risk of undesirable treatment effects on mechanisms regulating clinically relevant issues, such as vaccination responses and infection management in MS patients. As the knowledge of the DMT´s immunological impact has increased, the necessity of thorough immunological survey has been much acknowledged. We therefore performed a comprehensive mapping of absolute numbers and proportions of relevant cell populations in MS patients treated with fingolimod for one year.

## Materials and methods

### Study population

Fingolimod treatment (Gilenya ®, Novartis; 0.5 mg daily) was initiated in 19 patients with relapsing remitting MS, 12 women and 7 men (median age 45, range 26–50). Disease duration varied from 1.2–19 years (Table 1). Patients were consecutively recruited from the Department of Neurology at Linköping University Hospital between 2011 and 2015. No patient was treatment naïve, but five patients had at least a one-year treatment-free interval before inclusion. Nine patients had switched (eight because of JC virus serology conversion and one because of adverse effects) from natalizumab to fingolimod, after a 3-month washout period (except for one patient with a 2-month washout), and five patients switched from interferon-β because of treatment failure (Table 1). Blood samples from 18 healthy blood donors were used for baseline comparisons of the main lymphocyte populations (Table 1).

**Table 1:** Patient and healthy control (HC) characteristics at baseline.

	Patients	HC
No. of subjects	19	18
Median age (years)	45 (range 26–50)	42 (range 26–63)
Sex (Male/Female)	7/12	4/14
Median disease duration (years)	8.8 (range 1.2–19)	NA
EDSS (no. of subjects)		
0–3.5	17	NA
4.0–5.5	2	
6.0–9.5	0	
Median EDSS	1.5 (range 0–5)	NA
Previous treatment^a^		NA
Interferon-β	5	
Natalizumab	9	
No treatment	5	
Median number of relapses last two years before treatment	0 (range 0–3)	NA
No. of patients with relapse within one month before baseline	0	NA
Gadolinium-enhanced lesions at baseline (no. of patients)		
0 lesion	14	NA
1 lesion	3	
2 lesions	1	
3 lesions	1	
Median CSF total leukocyte count	0.8 (range 0.1–8.7) ^b^	ND
Median IgG index	0.66 (range 0.53–0.89) ^b^	ND
Median Albumin ratio	4.4 (range 2.3–7.5) ^b^	ND

EDSS = Expanded Disability Status Scale, n = 19 unless stated otherwise. Cerebrospinal fluid (CSF) was only collected at baseline, in a limited number of patients. ^a:^ disease-modifying treatment last year before baseline, b: n = 9, NA = not applicable; ND = not done.

All patients fulfilled the McDonald criteria of relapsing remitting MS [11]. MS patients were examined by a neurologist and scored according to the Expanded Disability Status Scale, Multiple Sclerosis Severity Scale and the Multiple Sclerosis Severity Score [12] (Table 2). The Symbol Digit Modalities Test (SDMT) [12] and the Multiple Sclerosis Impact Scale (MSIS-29) [13] were also performed. Brain MRI scans were performed at baseline and after one year of treatment with fingolimod. MRI was carried out in all patients according to a standardized MS protocol including gadolinium-enhanced contrast series.

**Table 2:** Clinical parameters at baseline and at follow-up after 1-year fingolimod treatment.

Clinical and CSF parameters	Baseline	1-year follow-up	p-value
EDSS	1.5 (0–5)	1.5 (0–6)	0.04
MSSS	1.92 (0.13–7.32)	2.01 (0.05–7.97)	0.29
MSIS-29			
Physical	1.7 (1.0–2.8)	1.5 (1.0–4.0) ^a^	0.78
Psychological	1.7 (1.1–3.9)	1.4 (1.0–3.6) ^a^	0.20
SDMT	55 (32–77)	57 (41–80) ^a^	0.008

Median values are given and range within parenthesis. n = 19 unless stated otherwise. p-value refers to related samples Wilcoxon signed rank test comparing baseline and follow-up. a: n = 18 because of lack of follow-up data. Abbreviations: EDSS = Expanded Disability Status Scale, MSSS = Multiple Sclerosis Severity Score, MSIS-29 = Multiple Sclerosis Impact Scale 29, SDMT = Symbol Digit Modalities Test.

Sampling of peripheral blood was obtained immediately before the start of fingolimod treatment in 19 patients. At follow-up, after one year (median 13 months, range 11–15), blood samples were obtained from all 19 patients. CSF samples were collected from nine of the patients at baseline. The study was approved by the regional ethics committee in Linköping (Dnr2012/405-31) and written consent was obtained from all participants.

### Definition of no evidence of disease activity (NEDA)

Patients were divided into two groups depending on the clinical and radiological outcome after 1-year treatment. Nine patients were categorized as showing NEDA at follow-up, corresponding to the concept first described by Havrdova *et al* [14]. The remaining ten patients were categorized as patients showing evidence of disease activity (EDA) and displayed at least one of the following during follow-up; a relapse (n = 3), new MRI activity (n = 5) or an increase in EDSS of 1.5 points from a baseline score of 0, of 1.0 point from a baseline score of at least 1.0, or of 0.5 points from a baseline score of greater than 5.0 (n = 4), detailed in Table 3.

**Table 3:** Characteristics of patients (n = 10) showing disease activity during follow-up (EDA).

Patient	Relapse on treatment	GD+ at one year	New lesion at one year	EDSS progression
1			x	x
2		x	x	
3			x	
4	x			x
5			x	
6				x
7	x			
8			x	
9				x
10	x			

### Flow cytometry

Whole blood was drawn in EDTA tubes and the cells were incubated with fluorochrome-conjugated antibodies against human CD3-FITC, HLA-DR-FITC, CD45RA-FITC, CD8-PE, CD16+56-PE, CD69-PE, CD28-PE, CD3-PerCP, CD45-PerCP, CD45RO-PerCP-Cy5.5, CD4-PE-Cy7, CD25-PE-Cy7, CD25-APC, CD39-APC, CD62L-APC, CD8-APC-H7, CD4-APC-H7, CD3-Horizon V450, CD56-Horizon V450, all from BD Biosciences (San Jose, USA). Anti-Foxp3-PE was purchased from eBioscience (San Diego, USA) and Helios-Pacific Blue from Biolegend (San Diego, USA). Lymphocyte subpopulations from peripheral blood were measured by 4- or 7-color combinations. TruCount^™^ tubes (BD Biosciences) were used for assessment of absolute numbers (cells/μl) of leukocytes (CD45), and major lymphocyte populations; T lymphocyte (CD3), T helper (CD4), T cytotoxic (CD8), B (CD19) and NK (CD16+CD56) cells, as described by the manufacturer. Absolute numbers of subpopulations were derived from the major populations. Proportions of major populations were given as % of lymphocytes and proportions of subpopulations were given as % of their parent population.

### Flow cytometry gating and analysis

Sample acquisition was performed on a FACSCanto II (BD Biosciences) flow cytometer with the FACSDiva software (BD Biosciences). Data analyses were performed with Kaluza software (version 1.3; Beckman Coulter, Miami, USA). Lymphocytes were identified by CD45 and side scatter (SSC) or forward scatter (FSC) and SSC. Differentiation status of T helper and cytotoxic T cells was determined with CD45RA and CD62L as shown in Fig 2. Treg cells were defined as naive (FOXP3dimCD45RA+) or memory (FOXP3brightCD45RA-) according to the gating strategy described by Miyara *et al* [15]. The FOXP3bright gate was set to include cells expressing the very highest levels of FOXP3, while FOXP3dim gated cells were slightly lower in FOXP3 expression but still positive.

### Statistical methods

For comparisons of flow cytometry data in healthy controls and in patients at baseline and at follow-up, paired samples t-test was performed. Clinical data was analyzed with related samples Wilcoxon signed rank test. Comparisons between the NEDA and EDA patient groups were performed with Mann-Whitney U-test, due to the low number of individuals in each group. Statistical significances were set at a two-tailed p-value of 0.05. All statistical analyses were performed in IBM SPSS Statistics 25 (SPSS inc., Chicago, IL, USA).

## Results

### A marked decline of lymphocyte populations with relative preservation of CD8+ cytotoxic T cells and NK cells

**Fig 1:**
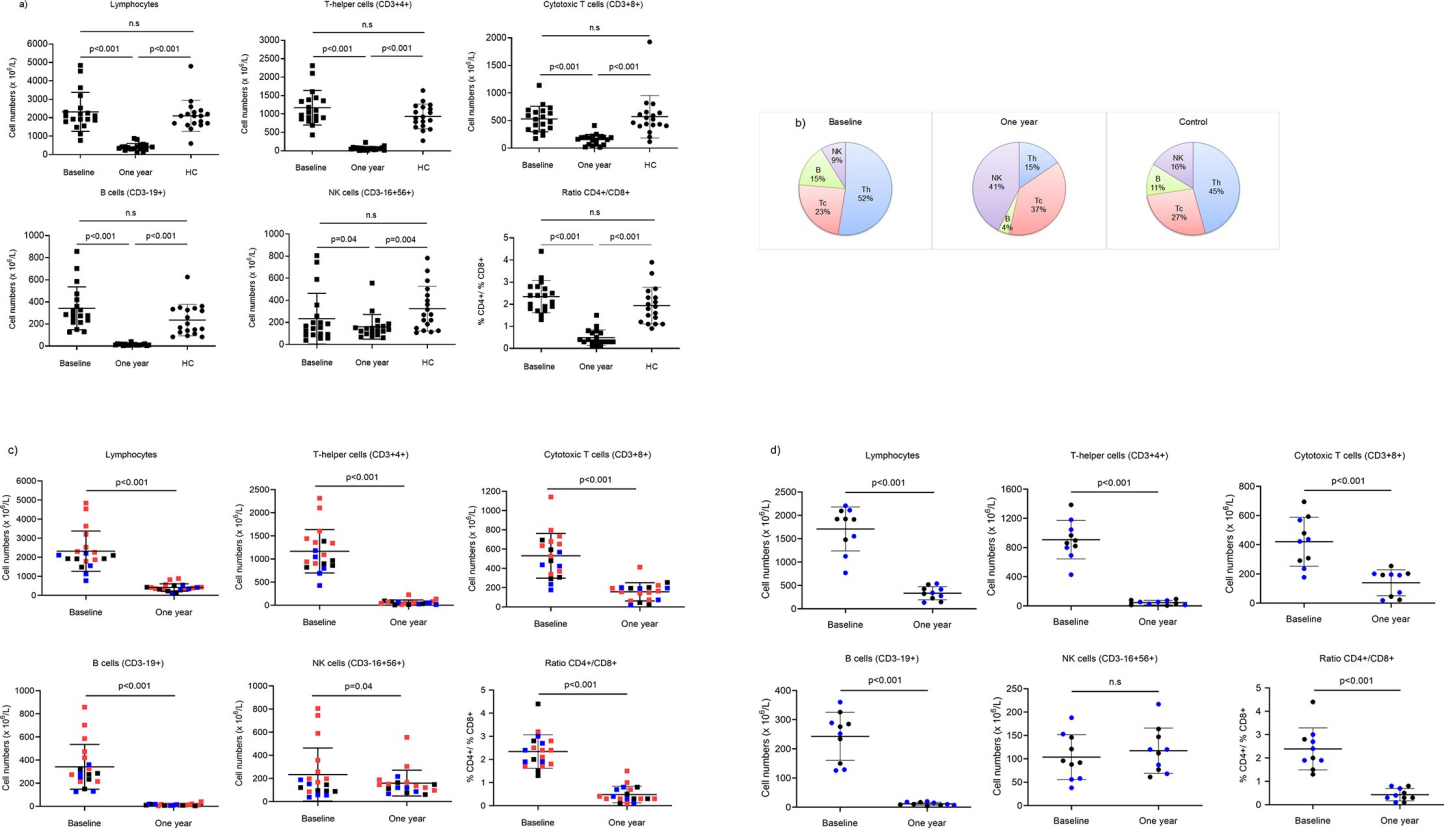
Distribution of lymphocytes in fingolimod treated patients and healthy controls. (a) Scatter plots showing the number (x10^6^/L) of lymphocytes, T helper (Th) cells, cytotoxic T (Tc) cells, B cells, NK cells and the ratio of CD4+/CD8+ (mean and SD) at baseline and after one year of fingolimod treatment in MS patients (n = 19) and in healthy controls (n = 18). (b) Pie charts illustrating the change in relative mean distribution of Th, Tc, B and NK cells at baseline, at the 1-year-follow up and in healthy controls. (c) Scatter plots highlighting the previous disease modifying treatment and the number of lymphocytes and subpopulations at baseline and after treatment. Red = natalizumab, blue = interferon-β, black = untreated. (d) Scatter plots excluding previously natalizumab-treated patients at baseline and after one year of fingolimod therapy.

Treatment with fingolimod for one year significantly reduced the total number of lymphocytes in peripheral blood of all patients. CD4+ Th and B cells were predominantly affected (p<0.001) (Fig 1A). The numbers of CD8+ cytotoxic T cells were also reduced (p<0.001) while the number of NK cells were only moderately affected (p = 0.04). The baseline numbers of all major lymphocyte subpopulations were comparable between MS patients and healthy control individuals (no significant differences were observed) (Fig 1A). The relative cell distribution, which is generally stable within individuals over time [16], was radically changed by the treatment, due to the predominant decline of Th cells and B cells. The mean proportion of Th cells was reduced from 52% to 15% (Fig 1B), while the reduction of cytotoxic T cells, not being so dramatic in absolute numbers, led to an increased proportion from 23% to 37%. Consequently, the CD4/CD8 ratio was reduced from 2.3 at baseline to 0.5 at follow-up. The dramatic decrease in B cells resulted in a reduction of the mean proportion from 15% to 3.7%, while the least affected population, NK cells, increased their proportion from 9% to 41% after one year of treatment. The CD56bright and CD56dim subpopulations were equally affected by treatment with preserved proportions (expressed as % of NK) after treatment; CD56bright and CD56dim cells constituted mean 8.7% and 91% before, and 7.2% and 93% after treatment, respectively. To investigate if the DMT´s before baseline influenced the distribution of the main lymphocyte populations, we highlighted the different treatments in a before-after plot in order to observe any patterns (Fig 1C). Patients previously treated with natalizumab, generally had the highest absolute counts for the total lymphocyte population, as well as for all the main subpopulations (Th cells, cytotoxic T cells, B cells and NK cells. However, the exclusion of patients previously treated with natalizumab did not change the pattern of a marked reduction of lymphocytes and its subpopulations after one year of fingolimod therapy (Fig 1D). The only exception from this was the NK-cell numbers, which were unchanged after treatment.

### Predominant depletion of naïve and central memory CD4+ and CD8+ T cells from blood

**Fig 2:**
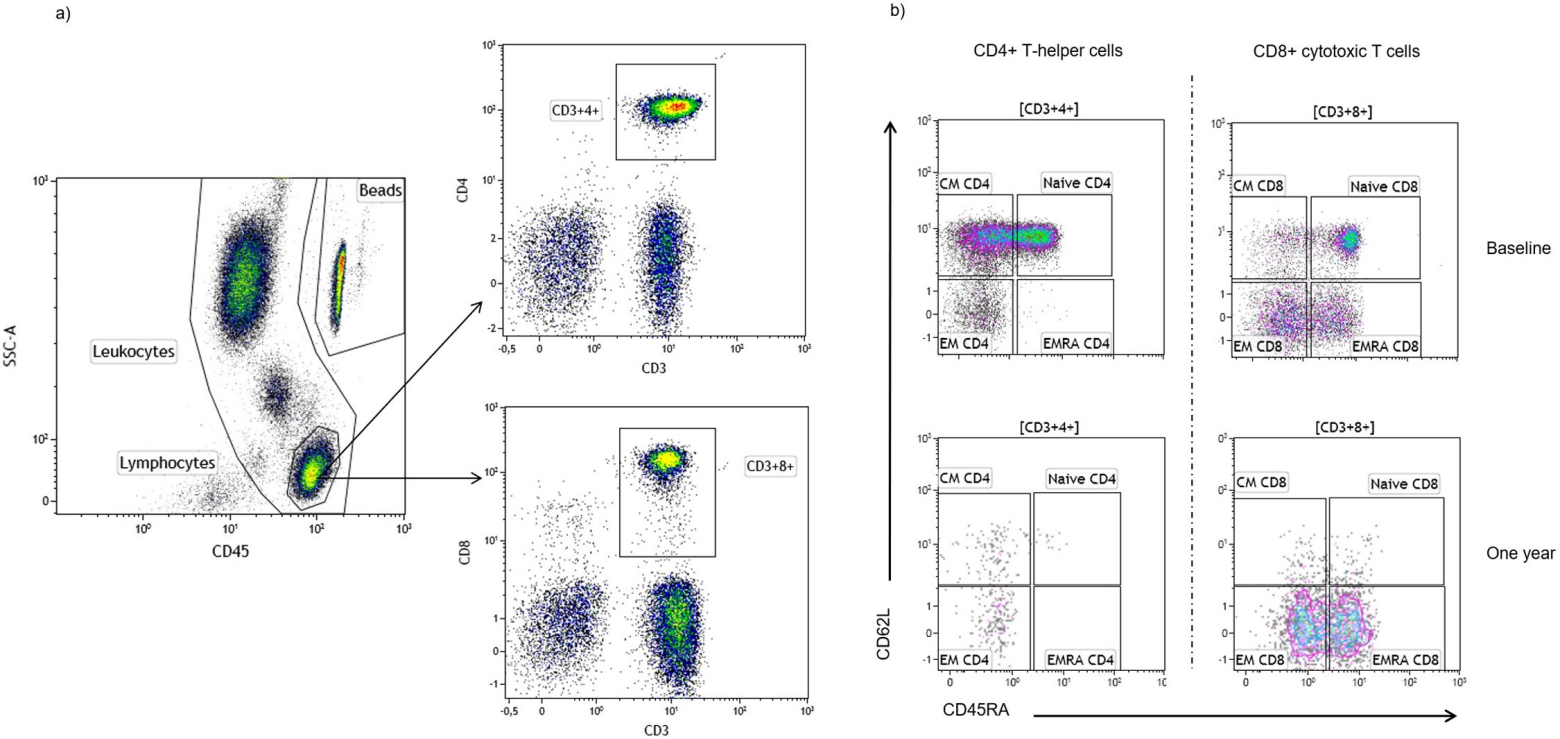
Gating strategy of lymphocytes and T-cell subpopulations. (a) Representative flow cytometric dot plots illustrating the gating strategy of lymphocytes and T cells (CD3+4+ and CD3+8+). (b) Representative T-cell differentiation pattern with respect to CD45RA and CD62L expression and the effect of fingolimod treatment in CD3+4+ (left column) and CD3+8+ cells (right column). The top panels indicate a typical distribution at baseline while the lower panels show the radical redistribution after one year of fingolimod treatment. Naïve cells were gated as positive for both CD45RA and the ligand for lymph node homing, CD62L; central memory (CM) as CD45RA-CD62L+; effector memory (EM) as CD45RA-CD62L- and terminally differentiated effector memory RA-positive cells (TEMRA) as CD45RA+CD62L-.

**Fig 3:**
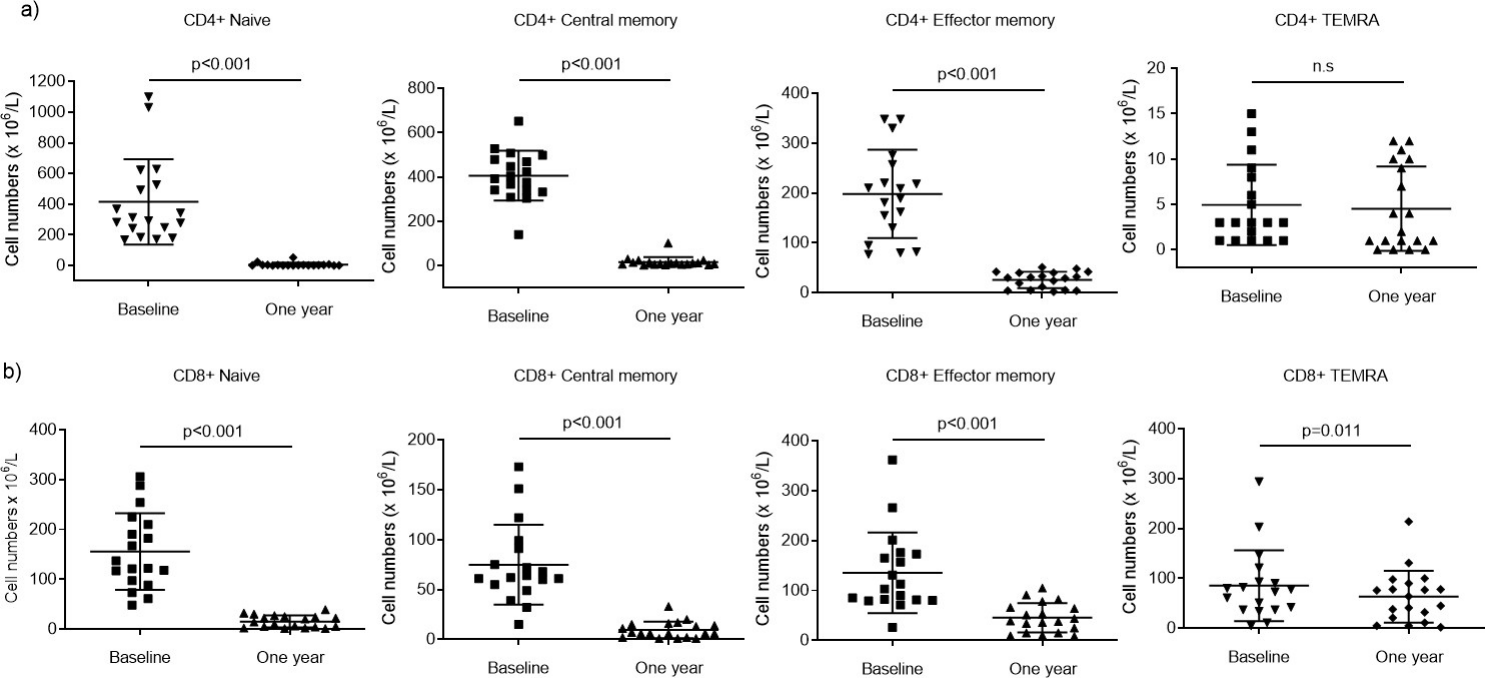
Effect of fingolimod on differentiation pattern of CD4+ and CD8+ cells. (a) Scatter plots showing the number (x10^6^/L) of CD4+ and (b) CD8+ cells (mean and SD) from patients (n = 18) at baseline and after 1-year of fingolimod treatment. (c) Pie charts illustrating the change in relative mean distribution of CD4+ and (d) CD8+ cells at baseline and after 1 year. Subpopulations are defined as naïve, central memory (CM), effector memory (EM) and terminally differentiated effector memory cells (TEMRA) depending on their expression of CD45RA and CD62L.

The selective effect of fingolimod was evident also when assessing subpopulations of CD3+ Th and cytotoxic T cells, illustrated in Fig 2A and 2B. The numbers of naïve and central memory (CM) Th cells were the most profoundly affected (Fig 3A; p<0.001), which is in line with the selective impact on cells circulating through the lymph nodes. The effector memory (EM) cells were also reduced (p<0.001), while the number of terminally differentiated effector memory cells (TEMRA) were more or less unaffected by fingolimod, which led to a radical redistribution of the subsets (Fig 3C and 3D). A similar pattern was seen for CD8+ T cell subsets (Fig 3B), although a lower decrease and sustained numbers of TEMRA was more evident among these cells.

### Increase in proportion of memory regulatory T cells

**Fig 4:**
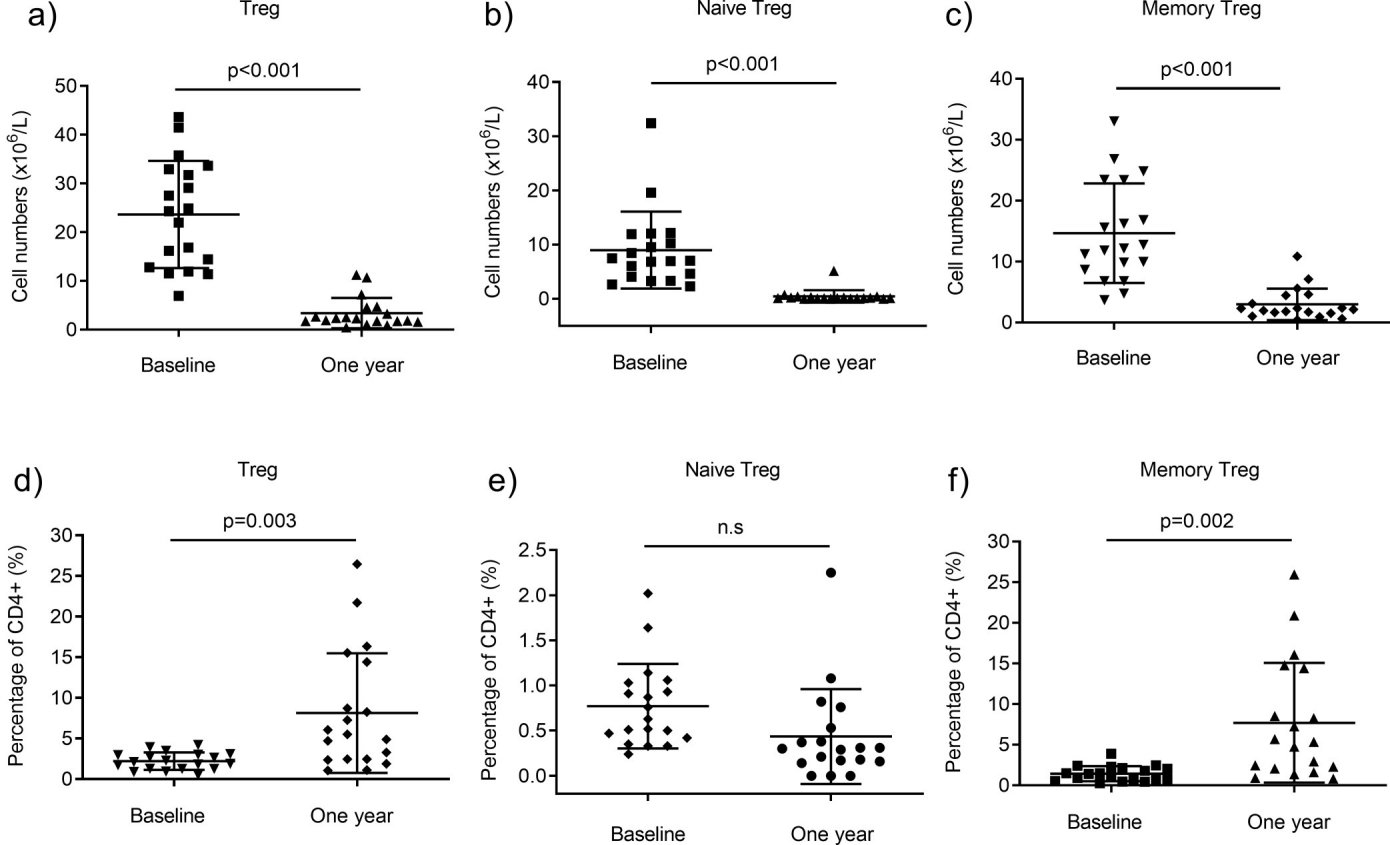
Effect of fingolimod on regulatory T cells (Tregs). (a) The number (x10^6^/L) of Tregs (sum of naive (FOXP3dimCD45RA+) and memory (FOXP3+CD45RA-)) at baseline and after 1-year fingolimod treatment (n = 19). (b) Both the naive and (c) memory Tregs were reduced in numbers. (d) The lower panel shows the percentage of Tregs at baseline and after one year; sum of naive and memory, (e) exclusively naive and (f) exclusively memory.

The number of regulatory T cells (Tregs) was reduced after one year of fingolimod treatment (Fig 4A; p<0.001). Both naive (FOXP3dimCD45RA+) and memory (FOXP3+CD45RA-) Treg cells decreased in numbers (Fig 4B and 4C; p<0.001). Notably, the Treg population in peripheral blood was more conserved as compared with the general decrease in total CD4+ cells, evident by the increased percentage after one year (p = 0.003; Fig 4D). Despite the dramatic reduction of the naïve CD4+ cell population, the percentage of naive Tregs (FOXP3dimCD45RA+) was relatively stable after one year. Furthermore, the proportion of memory Tregs (FOXP3+CD45RA-), increased at follow-up. This supports the finding of a relative preservation of the memory T-cell pool of CD4+ and CD8+ cells.

### Proportions of activated T and NK cells are affected by fingolimod

**Fig 5:**
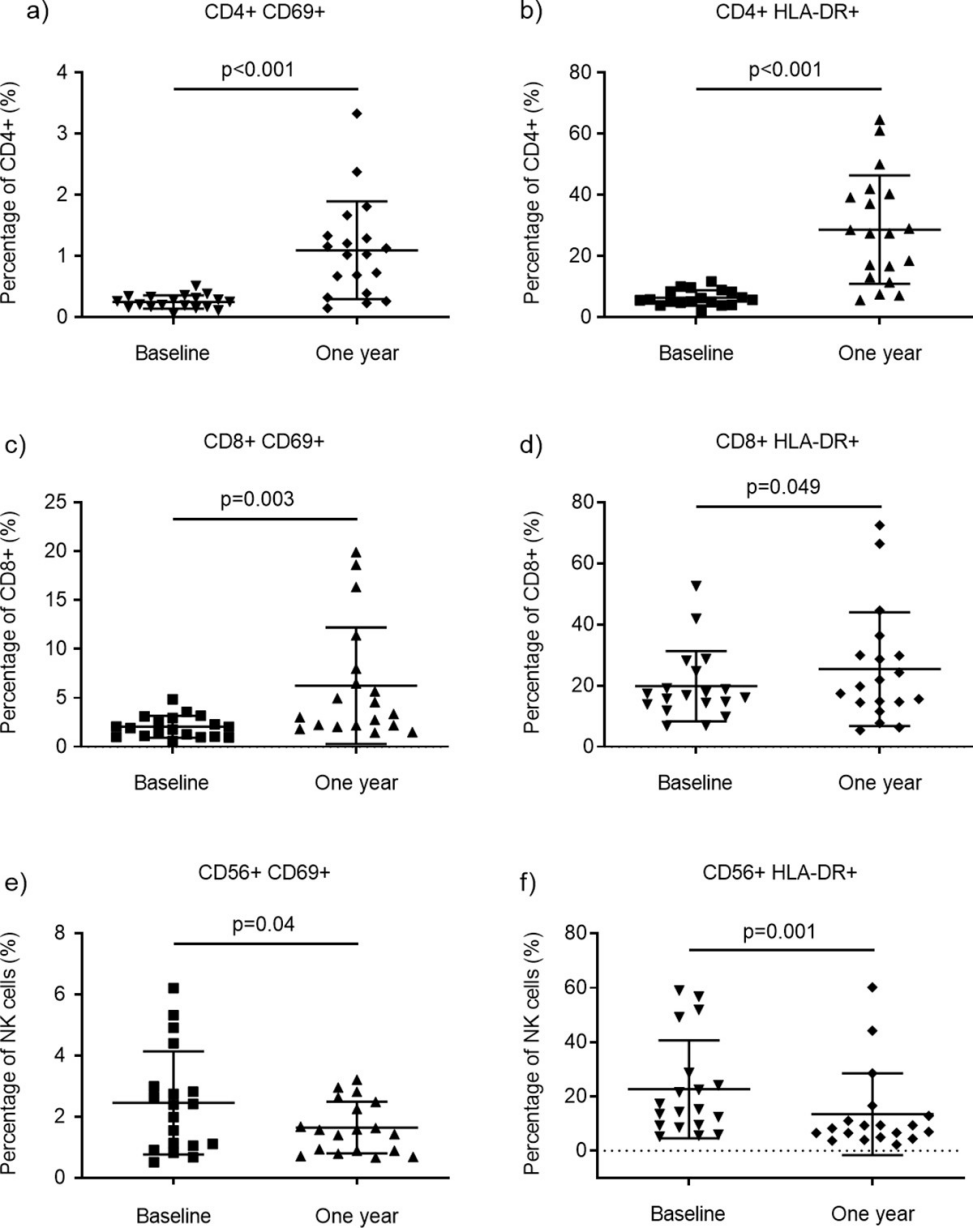
Effect of fingolimod on activation markers. (a) Scatter plots showing the percentage (mean and SD) of CD4+ cells expressing the activation markers CD69 and (b) HLA-DR in patients at baseline and after 1-year fingolimod treatment (n = 19). (c) CD69 and (d) HLA-DR expression in CD8+ cells and in (e-f) NK cells.

Significant changes in the proportions of activated T and NK cells were found after one year (Fig 5). The absolute numbers of CD69 and HLA-DR expressing T cells were reduced, but not as radically as for the Th and Tc cell populations in general, resulting in increased proportions of activated T cells after treatment (Fig 5A–5D). In contrast, the proportion of activated NK cells decreased after treatment (Fig 5E and 5F). Within the NK cell population, the proportion of HLA-DR+ CD56bright cells was stable at about 50%, while it slightly decreased for the CD56dim cells from 19% to 12% (p = 0.01) after one year (S1 Fig).

Regarding T cells expressing both CD4 and CD8, i.e. double positive T cells, as well as double negative T cells, the proportions were increased after one year of treatment (p = 0.001 and p<0.001, respectively), although the absolute numbers were decreased (S2 Fig).

### Patients with no evidence of disease activity (NEDA) have reduced proportions of activated Th and NK cells but higher numbers of TEMRA Th cells before treatment

**Fig 6:**
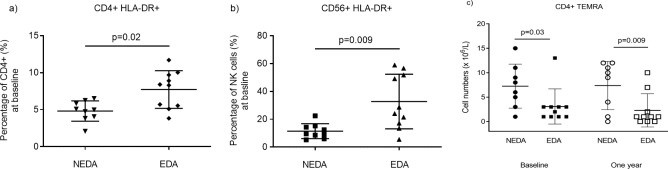
Differences in baseline characteristics of NEDA compared to EDA patients. (a) Scatter plots showing the baseline percentage of T helper and (b) NK cells expressing the activation marker HLA-DR (mean and SD) in patients with no evidence of disease activity (NEDA) and in patients with evidence of disease activity (EDA) (n = 19). (c) The number (x10^6^/L) of terminally differentiated effector memory (TEMRA) T helper cells at baseline and after one year in NEDA and EDA patients.

The annualized relapse rate (ARR) in the whole patient group was 0.15 during the follow-up period. Nine patients were categorized as showing NEDA at 1-year follow-up. The majority of these patients received natalizumab the last year before inclusion (distribution of treatment before inclusion in NEDA patients: 5 natalizumab, 1 interferon-β, 3 untreated). Few significant differences in immune profile between patients categorized as showing NEDA or not during fingolimod treatment were observed. A couple of potentially interesting findings were lower proportions of activated (HLA-DR+) Th and NK cells at baseline among NEDA patients, compared to patients showing disease activity during treatment (p = 0.02 and p = 0.009 respectively; Fig 6A and 6B). Furthermore, the number of terminally differentiated effector memory Th cells (TEMRA) was higher among NEDA patients, compared to patients showing disease activity during treatment both at baseline (p = 0.03) and after one year of treatment (p = 0.009) (Fig 6C).

## Discussion

In this one-year follow-up study of relapsing MS patients receiving fingolimod therapy, we report a significant reduction in lymphocytes, especially within the Th and B cell populations. The numbers of cytotoxic T cells were also decreased while NK cells were only moderately affected by the treatment, which led to a pronounced redistribution in blood of the proportion of the major lymphocyte populations. These findings are in line with previous studies, confirming the stipulated therapeutic action of fingolimod [17, 18]. However, to our knowledge, this study is the first to show a discrepancy between how the naïve and memory subgroups of Tregs are affected in count and proportion by fingolimod treatment.

Cells that preferentially circulate into lymph nodes, primarily Th and B cells, but also cytotoxic T cells while only to some extent NK cells, are trapped during fingolimod treatment leading to a dramatic decrease in peripheral lymphocyte numbers. We here wanted to further investigate the effect of fingolimod on subpopulations of T and NK cells. Predominantly naïve and CM numbers of Th and cytotoxic T cells were reduced, confirming previous findings showing that cells expressing the homing receptor CCR7 are the most heavily affected [19]. We used CD62L instead of CCR7 as a marker for lymphocyte homing since their expressions has been shown to be largely co-expressed and CD62L is suitable for staining of freshly prepared human lymphocytes [20, 21]. The numbers of EM cells were also lowered, but to a lesser extent, leading to a relative preservation of EM and TEMRA cells. These cells remain in the circulation and non-lymphoid organs since they are lacking CCR7/CD62L [22]. Probably the diminished peripheral supply of EM precursors, *i*.*e*. naïve and CM cells, caused the reduction in absolute numbers of EM cells. Further, we found a relative preservation of Treg cells as compared to the general Th cell population after one year of fingolimod treatment, confirming previous findings [17]. The majority of human Tregs lack expression of CCR7 and CD62L, which explains why they are not targeted by S1P mediated chemotaxis [23]. Our novel observation of a relative increase in memory Tregs during fingolimod therapy may be highly relevant since Tregs are considered as key immunomodulators with suppressive abilities in autoimmune responses [24]. In specific, it has previously been shown that fingolimod, by reducing numbers of CCR7-expressing Th cells, indirectly can reverse Treg dysfunction in MS patients [23].

We also report a slight decrease in absolute numbers of NK cells. Since this population was not as dramatically affected, its proportion increased compared to other lymphocytes. NK cells normally circulate in peripheral blood, but rapidly move to sites of inflammation in peripheral tissues, due to gradients of chemokines. The majority of NK cells (CD56dim) do not traffic secondary lymphoid organs since they lack expression of CCR7 and CD62L [25]. However, CD16- NK cells (CD56bright) express high levels of these receptors, and respond to CCR7 and CD62L ligands, which suggests that these cells are targeted by fingolimod [25]. The egress of NK cells from lymph nodes might be regulated through both S1P1 and S1P5 receptors [26, 27] whereas the rest of the lymphocytes depend mainly on the S1P1 receptor [28]. The CD56dim cells have been shown to have a higher expression of S1P5 than the CD56bright NK cells, which in theory would lead to a relative increase in CD56dim. However, we found that the fractions of CD56dim and CD56bright were equally affected, which might suggest alternate SIP receptors or other factors regulating NK cell lymph node trafficking [29, 30].

In order to evaluate if lymphocyte subpopulation changes during fingolimod treatment were reflected in clinical and radiological outcome, we divided patients into two groups depending on whether disease activity during the 1-year follow-up was detected or not. We used the concept of no evidence of disease activity, based on the lack of new relapses, no MRI activity and no confirmed disability measured as progression in EDSS (NEDA-3). About 50% of the patients did not fulfill the criteria of NEDA which, at first sight, is a high number. However, when comparing to real-world data on these outcome variables during fingolimod treatment, similar data has been found. Izquierdo *et al* report an absolute relapse rate (ARR) of 0.3 during fingolimod treatment and a proportion of patients free of new or enlarged T2-weighted lesions of 69% after one year of treatment [31]. In our cohort the ARR was 0.15 and number of patients free from MRI activity after one year was 14 out of 19 (74%). Four patients showed confirmed disability progression at follow-up in our cohort, representing a cumulative disability progression of 21% after one year. The corresponding proportion of cumulative disability progression after one year of fingolimod treatment as reported by Izquierdo *et al*, was 6.9%. An explanation to this 3-fold difference could be that patients included in our cohort were older (mean age 41 years versus 37 years). To sum up, we state that the patient clinical outcome in this study in general, is in accordance with previously presented real-world data.

When we applied the NEDA concept, to analyze if the clinical and radiological outcomes also were reflected in differences in lymphocyte subpopulation changes during treatment, there were only a few findings. Although there are known alterations in several T-cell subsets during fingolimod treatment, only the CM Th cell population has been shown to be associated with MS relapse during this therapy [17]. It was suggested that relapsing patients had a higher inflammatory disease activity due to an insufficient decrease in CM [17]. In our data, we did not find any difference in the CM population when comparing patients with disease activity or not during follow-up. However, we did observe that individuals with no disease activity during treatment had higher numbers of TEMRA Th cells, both at baseline and after one year of treatment, compared to patients with disease activity. Because of the small number of patients in this study one cannot make any firm conclusions from this observation. Principally, this finding is interesting since it implies that patient peripheral lymphocyte profile pre-treatment may be of importance to predict treatment response of fingolimod therapy. Another finding that potentially could be of clinical importance was that NEDA patients had lower proportions of Th and NK cells with an activated phenotype (HLA-DR+) before starting fingolimod therapy. This observation may reflect a difference in the baseline disease activity, i.e. that these individuals had a less aggressive MS disease before treatment started and accordingly had less propensity for relapses and developing new MR lesions during treatment, compared to patients with increased proportions of activated Th and NK cells.

The main limitations of this study are the relative low number of patients included and that the patients were not treatment naïve before inclusion. To address this, we aimed to describe these patients both in relation to the normal peripheral blood conditions and to possible effects caused by the previously used DMT´s. In that way, we consider it possible to evaluate the representativeness of this cohort and the relevance of our findings, despite the limited patient number. Starting with the comparisons between the normal peripheral blood lymphocyte conditions, we here used healthy blood donors as controls. No differences in numbers of the main lymphocyte subpopulations were observed between MS patients at baseline and controls. In contrast, after treatment there was an overall significant decline in numbers of lymphocytes in MS patients compared to controls. This observation is in line with earlier studies and furthermore, our additional main results are consistent and correspond well to previous reports on the effects of fingolimod on peripheral blood lymphocyte subpopulations. This implicates that also the novel findings we present are representative for the included patient characteristics. Since our aim was to describe the *difference* in numbers and distribution of lymphocyte subpopulations during fingolimod treatment, controls were not followed longitudinally. Instead, statistical analyses were performed using pairwise comparisons of lymphocyte subpopulation changes for each patient. In this respect each patient constituted their own control. Considering the known marked effects of fingolimod on numbers and distribution of lymphocytes in peripheral blood, we argue that it is most unlikely that the observed findings in the present study would be unrelated to the direct effects of fingolimod on lymphocyte subpopulations in peripheral blood. Nevertheless, our observation such as the relative preservation of memory Tregs after treatment should be confirmed in larger sample studies.

The patients in this cohort were not treatment naïve (although five patients were untreated for at least one year before inclusion). The DMT´s given before fingolimod are therefore potential confounders, which may have influenced the pattern of changes in peripheral blood lymphocyte compositions, thereby possibly obfuscating the effects of fingolimod. Yet, switching therapy between different DMT´s is often necessary in a clinical context (for example because of adverse effects). In that way, the present study may provide valuable information about how the peripheral blood lymphocyte composition is affected in such a clinical situation (switching from interferon-β or natalizumab to fingolimod). To control for possible effects of the previous treatments we performed before-after plots to detect any patterns in the change of lymphocyte subpopulations that could be related to the earlier DMT. We found that patients treated with natalizumab before baseline (despite a 3-month washout period) in general had higher lymphocyte counts compared to patients with other treatments, a finding in accordance with the known inhibiting effect of natalizumab on lymphocyte migration over the blood-brain barrier. However, when we excluded patients previously treated with natalizumab from the analyses, we still observed the same pattern of a marked reduction in numbers of all main lymphocyte subpopulations after one year of fingolimod treatment (the only exception for NK-cells). This observed significant reduction could therefore not depend merely on the withdrawal of natalizumab but must be related to the fingolimod treatment. Another aspect is that both clinical experience and previous studies [32, 33] describe the risk of recurrence of disease activity after the interruption of natalizumab treatment. As soon as two months after interruption, disease activity can be detected, measured as new MRI lesions. Plavina *et al* demonstrate that the effects of natalizumab on peripheral immune cells and other markers begin to decline starting at weeks 8–12, with levels returning to those observed or expected for patients not treated with natalizumab around 16 weeks after the last dose [32]. For this reason, a wash-out period longer than three months after natalizumab treatment, was not acceptable from a clinical point of view.

## Conclusion

We confirm that fingolimod treatment exerts marked effects on both lymphocyte absolute numbers as well as their proportions in peripheral blood of MS patients. However, considering the effect of fingolimod on the relative distribution of lymphocyte subsets, such as regulatory T cells, the clinical benefit of this therapy may not only result from an overall lymphopenia but could also be related to specific alterations in the distribution of immunoregulatory lymphocyte subsets. Our findings indicate that even though fingolimod treatment profoundly reduces the number of peripheral memory lymphocytes, significant parts of the immunological memory seem to be preserved.

## Supporting information

S1 Fig HLA-DR expression in CD56dim and CD56bright cells. (a) Scatter plots showing the percentage of HLA-DR positive cells within CD56dim and (b) CD56bright cells from patients at baseline and after 1-year fingolimod treatment.
(TIF)

S2 Fig Number and percentage of CD4+8+ and CD4-8- T cells. (a-b) Scatter plots showing the number (x10^6^/L) and (c-d) percentage of CD4+8+ T cells (left column) and CD4-8- T cells (right column) from patients at baseline and after 1-year fingolimod treatment.
(TIF)
