# Supplementary figures and images for: Treatment effects of fingolimod in multiple sclerosis: Selective changes in peripheral blood lymphocyte subsets
Source: PLoS One. 2020 Feb 3;15(2):e0228380. doi: 10.1371/journal.pone.0228380 (PMC6996838; doi:10.1371/journal.pone.0228380)

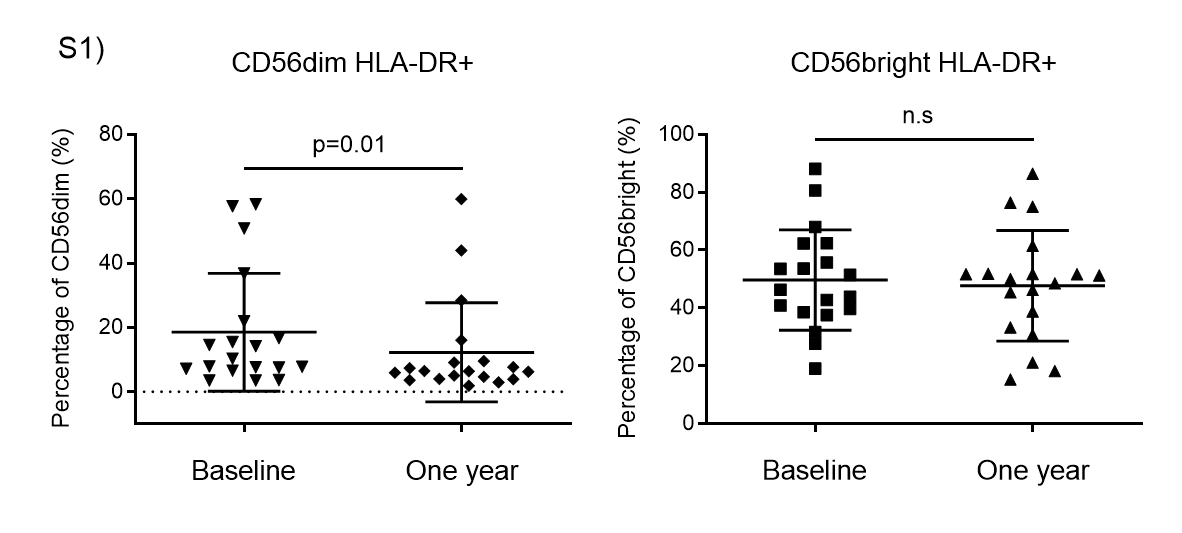

Supplement: S1 Fig — (a) Scatter plots showing the percentage of HLA-DR positive cells within CD56dim and (b) CD56bright cells from patients at baseline and after 1-year fingolimod treatment. (TIF) [file pone.0228380.s001.tif]

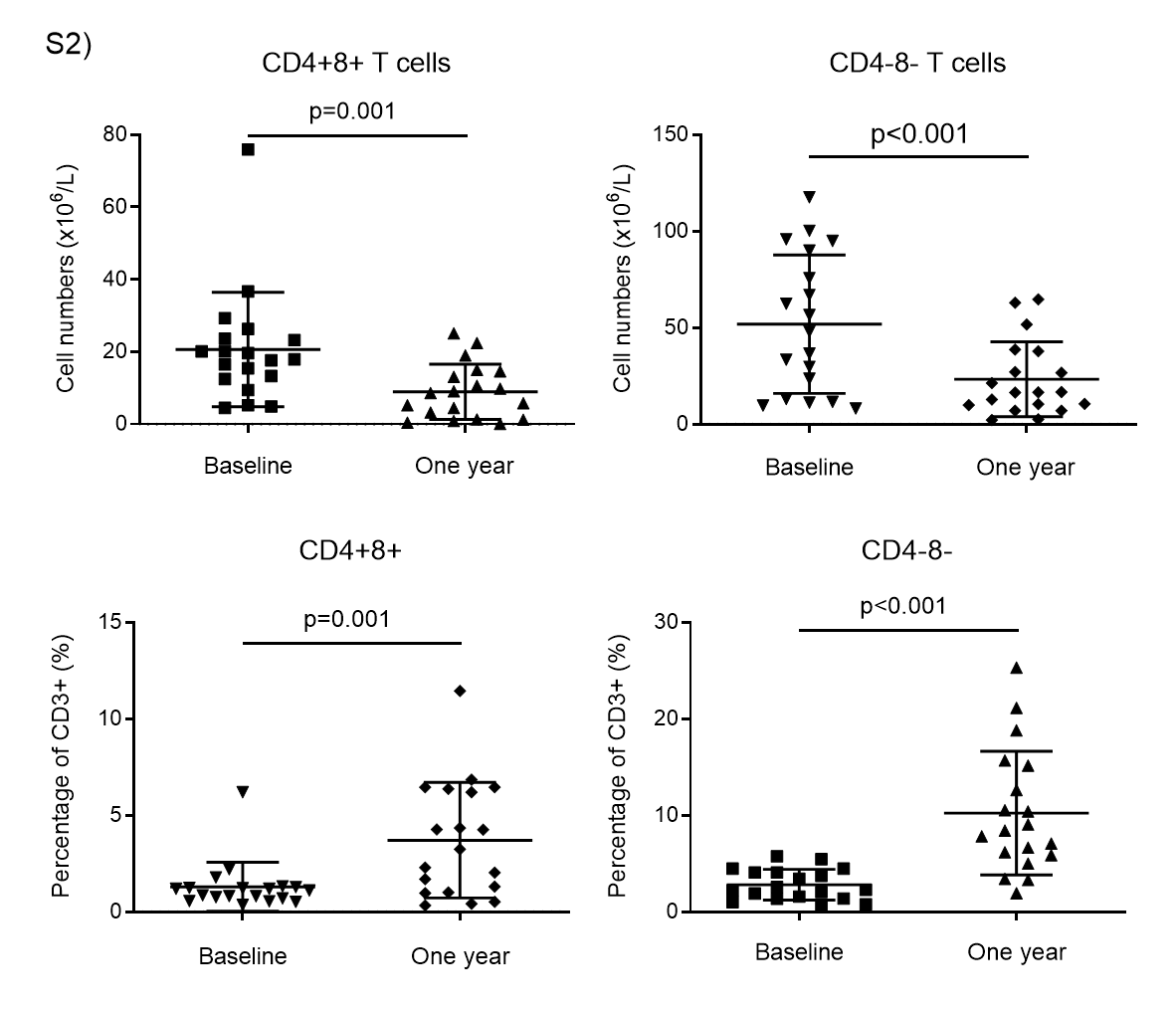

Supplement: S2 Fig — (a-b) Scatter plots showing the number (x106/L) and (c-d) percentage of CD4+8+ T cells (left column) and CD4-8- T cells (right column) from patients at baseline and after 1-year fingolimod treatment. (TIF) [file pone.0228380.s002.tif]
